# A labeled RF signal dataset for UAV detection and classification in the 2.4GHz band under Wifi and Bluetooth coexistence

**DOI:** 10.1016/j.dib.2026.113127

**Published:** 2026-08-05

**Authors:** Saber Mgannem, Radhoine Aloui, Bilel Hamdi, Sofien Mhatli, Ignacio Llamas-Garro

**Affiliations:** a SERCOM-Lab, École Polytechnique de Tunisie (EPT), Université de Carthage, La Marsa, Tunis, 2078, Tunisia; b Research Laboratory Smart Electricity ICT, SEICT, LR18ES44, National Engineering School of Carthage, University of Carthage, Tunisia; c Laboratory of the Communication Systems Sys'Com (LR-99-ES21), National Engineering School of Tunis, University of Tunis El Manar, BP.37 Le Bélvédère, 1002, Tunis, Tunisia; d Centre Tecnològic de Telecomunicacions de Catalunya (CTTC/CERCA), 08860, Castelldefels, Spain

**Keywords:** Dataset, Data article, Unmanned aerial vehicle (UAV), Radio frequency (RF), Spectrogram analysis, Object detection, Deep learning, ISM band

## Abstract

This article presents a labeled radio frequency (RF) dataset for the detection and classification of unmanned aerial vehicles (UAVs) operating in the 2.4 GHz industrial, scientific, and medical (ISM) band. The dataset was acquired using a USRP B210 software-defined radio equipped with bidirectional antennas in an indoor and semi-controlled wireless environment containing realistic WiFi and Bluetooth interference. Data acquisition was performed under multiple operational scenarios, including drone idle, take-off, hovering, movement, and absence of UAV activity. The collected RF signals were transformed into time–frequency spectrogram images using the Short-Time Fourier Transform (STFT) to support machine learning and deep learning applications [1]

The dataset was annotated using LabelImg [2] in a YOLO-compatible format to support object detection and classification tasks. This resource is intended for researchers working on RF-based drone detection, wireless spectrum monitoring [3], and deep learning-based signal classification. The dataset enables benchmarking under realistic interference conditions and supports reproducible research in UAV detection systems.

Specifications Table

**Table:** 

Subject	Computer Science / Wireless Communications
Specific subject area	RF-based UAV detection, spectrum sensing, and machine learning
Type of data	Images (.png), Text labels (.txt), Table
Data collection	RF signals were captured using a USRP B210 software-defined radio (SDR) equipped with an external bidirectional antenna. Acquisitions were performed with a center frequency of 2.438 GHz, a sampling rate of 56 MS/s, and a receiver gain of 45 dB. Signals were captured in an indoor and semi-controlled environment containing real WiFi and Bluetooth traffic alongside a DJI Phantom 4 Pro platform. Raw complex IQ data blocks of 1100,000 samples were transformed via STFT (NFFT = 512, overlap = 256) into images.
Data source location	Science and Technology for Defense Lab (STD), Military Research Center, Tunis, Tunisia
Data accessibility	Repository name:Mendeley Data Data identification number: 10.17632/y4w96kzsb5.1 Direct URL to data:10.17632/y4w96kzsb5.1 Instructions for accessing these data: The dataset is publicly available on Mendeley Data at: https://doi.org/10.17632/y4w96kzsb5.1 Users can access the data by: 1. Clicking the direct URL above or navigating to Mendeley Data 2. Downloading the complete dataset as a ZIP archive 1. Extracting the archive to access: ○ Training dataset (5678 spectrogram images with corresponding YOLO-format label files) ○ Validation dataset (1164 spectrogram images with corresponding YOLO-format label files) ○ A README file containing detailed instructions for dataset usage and reproduction For users with Mendeley Data accounts, the dataset can also be added to personal collections or cited directly through the provided DOI.

## 1 Value of the Data

• Addressing the Annotation Bottleneck:This dataset provides 6842 high-resolution, manually annotated spectrograms. By providing pre-labeled data in a YOLO-compatible format, this resource eliminates the labor-intensive manual labeling phase for researchers developing RF-based object detection frameworks [[1], [2], [3]].
• Realistic Spectral Coexistence: Unlike datasets captured in isolated environments, this data includes the intentional overlapping of UAV, WiFi, and Bluetooth signals. This allows for the development of ``interference-aware'' deep learning models capable of distinguishing drones from common ISM-band traffic [5].
• Hardware Invariance and Robustness: The inclusion of data captured across multiple sampling bandwidths (30, 40, and 56 MHz) enables researchers to train models that are robust to varying hardware constraints and sampling rates, improving cross-device generalization.
• Benchmarking and Reproducibility: The standardized STFT processing chain and labeling protocol allow for direct, reproducible performance comparisons with established benchmarks such as DroneRF [6] and CardRF [7], facilitating the tracking of state-of-the-art progress in UAV surveillance.
• Cross-Disciplinary Utility: This dataset is highly valuable for researchers in diverse fields, including wireless communications for spectrum sensing, cybersecurity for unauthorized drone mitigation, and electronic warfare for signal primitive identification [4].

## 2 Background

The compilation of this dataset was motivated by the critical need for robust, interference-resilient drone detection mechanisms in urban environments that share crowded wireless spectrum bands. Because commercial UAVs predominantly operate within the 2.4 GHz ISM band, separating drone control and video downlink transmissions from standard consumer traffic (such as high-density WiFi routers and frequency-hopping Bluetooth devices) presents a significant technical challenge. Traditional datasets often isolate drone signals in noise-free settings, failing to represent actual deployment environments. This dataset establishes a realistic multi-signal coexistence benchmark to train computer vision models to reliably detect drones under complex overlapping signal patterns.

## 3 Data Description

This article presents a labeled RF spectrogram dataset designed for commercial UAV detection in the 2.4 GHz ISM band under realistic wireless interference conditions. The dataset consists of 6842 spectrogram images, with a total of 22,375 annotated signal instances.

The detailed composition of the dataset across training and validation splits is provided in Table 1.

**Table 1:** Summary of the RF dataset composition and labeling statistics across train and validation splits.

Subset	Images	Drone	WiFi	Bluetooth	Total Labels
Training	5678	8454	3330	6771	18,555
Validation	1164	1654	693	1473	3820
Total	6842	10,108	4023	8244	22,375

*Note: The number of labels exceeds the number of images due to the concurrent presence of multiple wireless signals in the same time-frequency spectrogram.

The dataset considers three primary signal classes: Drone_Signal, WiFi, and Bluetooth. All spectrograms were manually annotated at the object level using bounding boxes provided in YOLO-compatible format.

As highlighted in Table 1, the dataset exhibits a high label density, with an average of approximately 3.27 signal instances per spectrogram. This high-density labeling reflects the realistic nature of the captured environment, where dense Bluetooth frequency-hopping activity and persistent UAV video links frequently coexist within the same spectral window.

## 4 Experimental Design, Materials and Methods

### 4.1 Hardware setup

**Fig. 1:**
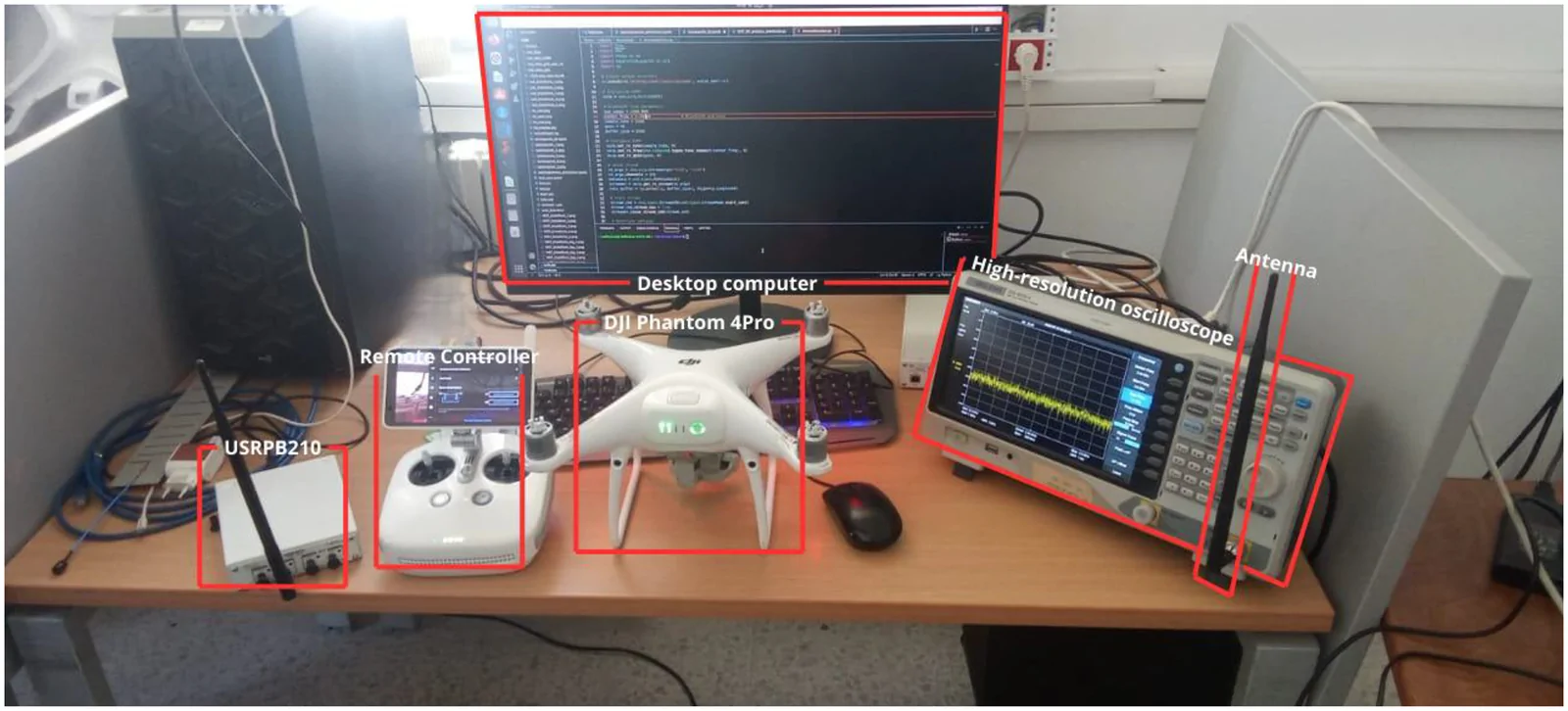
Experimental hardware setup used for RF signal acquisition. The setup includes a USRP B210 software-defined radio connected to an antenna for signal capture in the 2.4 GHz ISM band, a DJI Phantom 4 Pro commercial UAV with its remote controller, a desktop computer used for signal acquisition and processing, and a high-resolution oscilloscope for spectrum monitoring and signal verification.

Fig. 1. Experimental hardware setup used for RF signal acquisition. The setup includes a USRP B210 software-defined radio connected to an antenna for signal capture in the 2.4 GHz ISM band, a DJI Phantom 4 Pro commercial UAV with its remote controller, a desktop computer used for signal acquisition and processing, and a high-resolution oscilloscope for spectrum monitoring and signal verification.

The experimental setup used for RF data acquisition is illustrated in Fig. 1. The setup consists of a USRP B210 software-defined radio (SDR) connected to an external antenna for capturing RF emissions in the 2.4 GHz ISM band. A DJI Phantom 4 Pro commercial UAV and its remote controller were used as the primary signal source during different operational scenarios, including idle mode, take-off, hovering, and maneuvering. A desktop computer was employed for real-time signal acquisition, storage, and preprocessing, including IQ sample extraction and spectrogram generation. Additionally, a high-resolution oscilloscope was used to monitor the signal spectrum and validate the captured RF activity during the acquisition campaign.

The acquisition parameters were fixed as follows:

• Center frequency: 2.438 GHz
• Sampling rate: 56 MS/s
• Receiver gain: 45 dB
• Number of samples per acquisition: 1100,000

The selected center frequency corresponds to the operational frequency band of the DJI Phantom 4 Pro remote communication link and overlaps with the WiFi/Bluetooth coexistence band, allowing realistic interference-aware data collection.

### 4.2 Experimental environment

The data collection was conducted in an indoor and semi-controlled wireless environment where multiple RF sources were simultaneously active.

Three types of signals were intentionally captured:

• UAV communication signals from the DJI Phantom 4 Pro
• WiFi traffic in the 2.4 GHz band
• Bluetooth device transmissions

This configuration was designed to simulate realistic spectrum occupancy conditions encountered in practical drone surveillance scenarios.

### 4.3 Wireless interference signal characterization

To characterize realistic wireless interference conditions, additional spectrograms were generated for WiFi and Bluetooth signals under both idle and active transmission states.

**Fig. 2:**
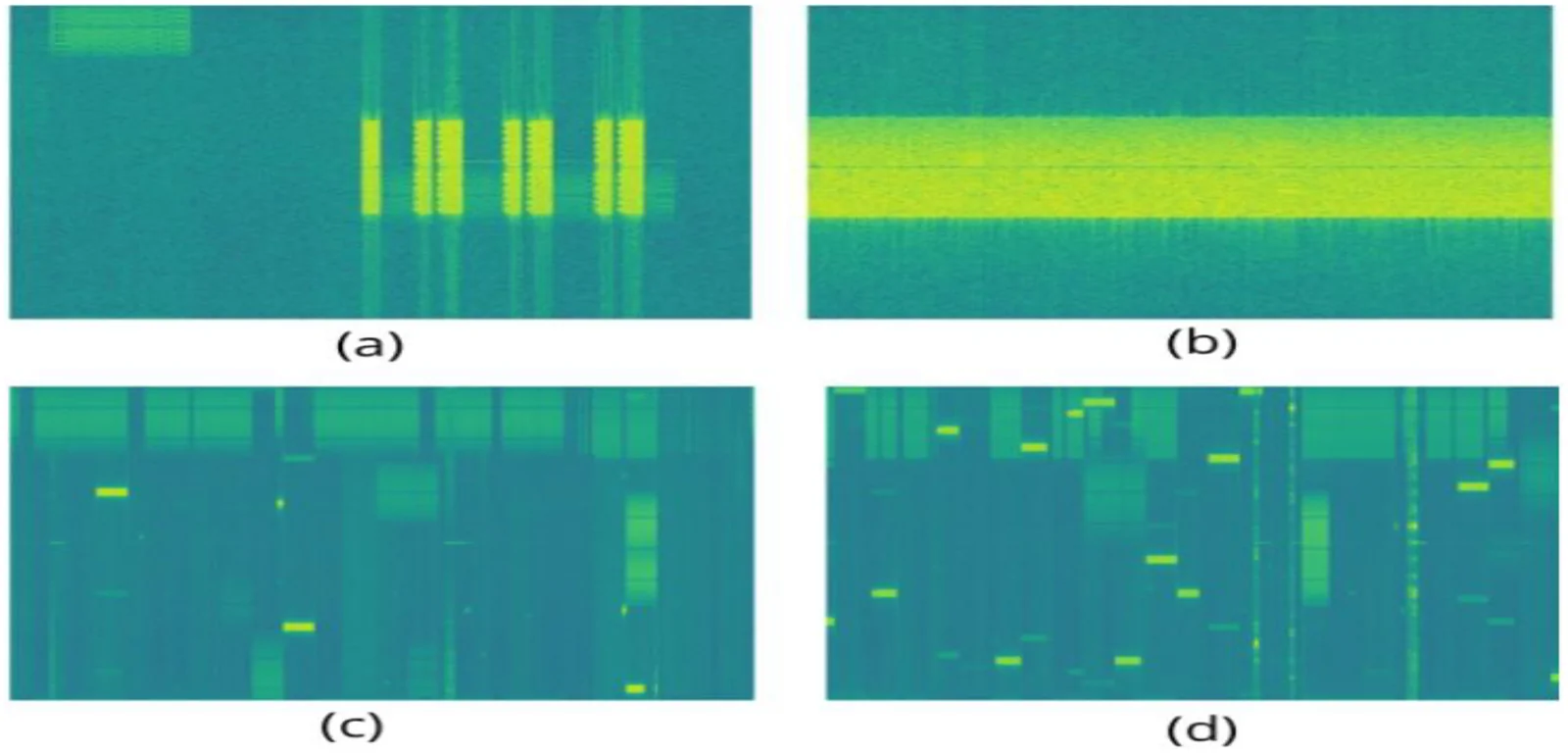
Representative spectrograms of WiFi and Bluetooth signals under different traffic conditions: (a) WiFi idle state, (b) WiFi active transmission, (c) Bluetooth idle state, and (d) Bluetooth active state during frequency-hopping communication.

For WiFi signals, the idle condition corresponds to a device associated with an access point without active payload transmission. In this case, the spectrum is mainly composed of periodic beacon and management frames, which appear as sparse and intermittent bursts in the time–frequency domain, as shown in Fig. 2(a).

During active transmission, continuous OFDM packets occupy the full communication bandwidth, producing a dense and sustained wideband spectral block, as illustrated in Fig. 2(b).

Bluetooth signals exhibit a distinct spectral morphology due to their frequency-hopping spread spectrum (FHSS) modulation. In idle mode, only occasional control and synchronization packets are transmitted, resulting in isolated narrowband bursts across hopping channels, as shown in Fig. 2(c).

Under active transmission conditions, such as audio streaming, the hopping activity becomes significantly denser, producing a rapid sequence of narrowband bursts distributed across the 2.4 GHz band, as shown in Fig. 2(d).

These examples highlight the variability of interference signatures under different traffic loads and justify the inclusion of realistic coexistence scenarios in the proposed dataset.

### 4.4 Drone signal spectral characteristics during active video transmission

In the present dataset, the UAV was operated in active video-streaming mode using the DJI Phantom 4 Pro platform. During this operational state, the RF emissions captured in the 2.4 GHz ISM band are primarily dominated by the continuous downlink communication established between the drone and the remote controller for first-person-view (FPV) video feedback.

Compared with WiFi and Bluetooth interference signals, the drone video transmission exhibits a distinctive spectral morphology. While active WiFi traffic may also occupy a relatively wideband channel, its temporal structure is generally packet-driven and depends on network traffic load. In contrast, the UAV video downlink presents a more persistent and temporally stable wideband spectral occupancy, reflecting the continuous nature of real-time video streaming and camera feedback transmission.

**Fig. 3:**
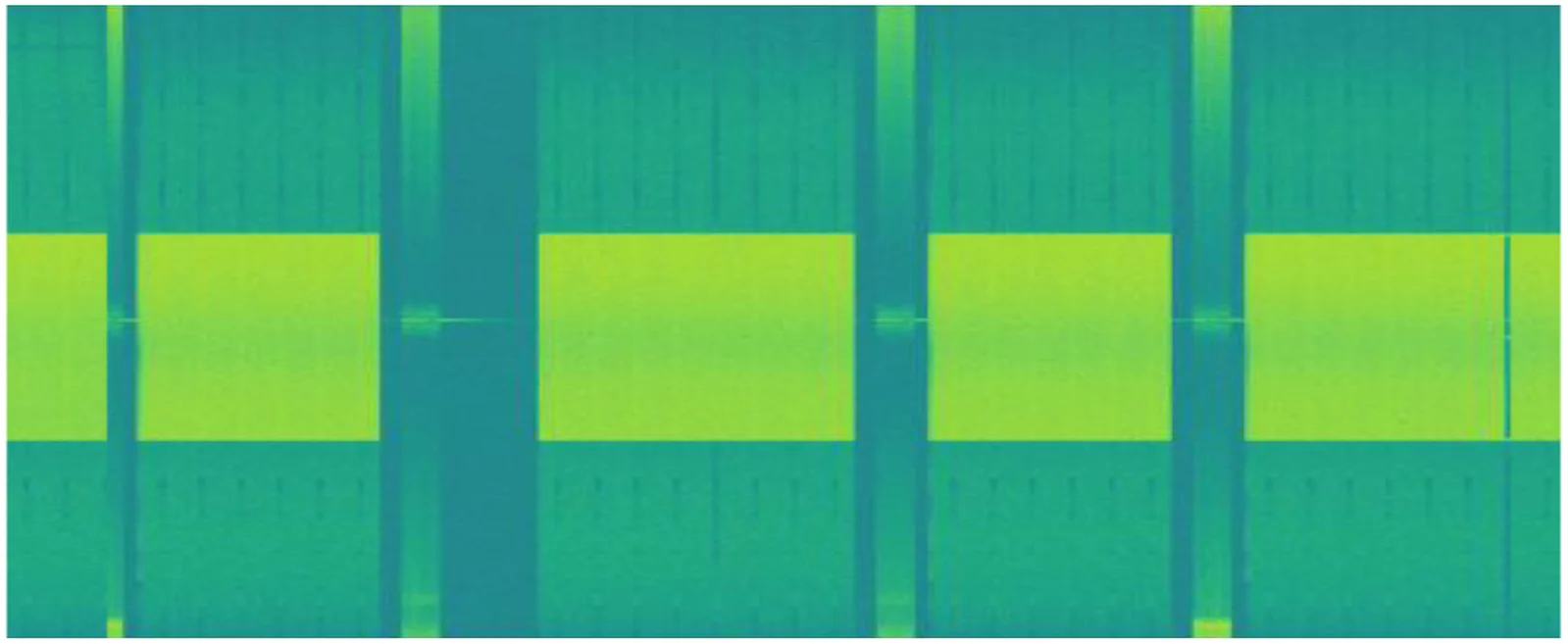
Representative spectrogram of the DJI Phantom 4 Pro during active video-streaming transmission in the 2.4 GHz ISM band, showing sustained wideband spectral occupancy and strong temporal continuity.

As illustrated in Fig. 3, the resulting spectrogram signatures typically appear as dense wide-band blocks with strong temporal continuity, making them visually distinguishable from sparse WiFi beacon activity and narrow-band Bluetooth frequency-hopping bursts.

This acquisition scenario was intentionally selected because active video transmission represents one of the most operationally relevant conditions for practical UAV surveillance, unauthorized drone detection, and spectrum monitoring applications.

### 4.5 Comparative analysis of indoor and outdoor spectral environments

To enhance the diversity and robustness of the dataset, RF signal acquisition was extended from the semi-controlled indoor laboratory to an uncontrolled outdoor environment. This transition introduces significant variability in the spectral signatures of the three target classes (Drone, WiFi, and Bluetooth).

In the indoor environment (described in the preceding subsections), the captured spectrograms exhibit high Signal-to-Noise Ratio (SNR) and a relatively “clean” background. The boundaries of the WiFi OFDM blocks and the Bluetooth FHSS bursts are sharp and clearly defined against the noise floor, as seen in Fig. 2.

**Fig. 4:**
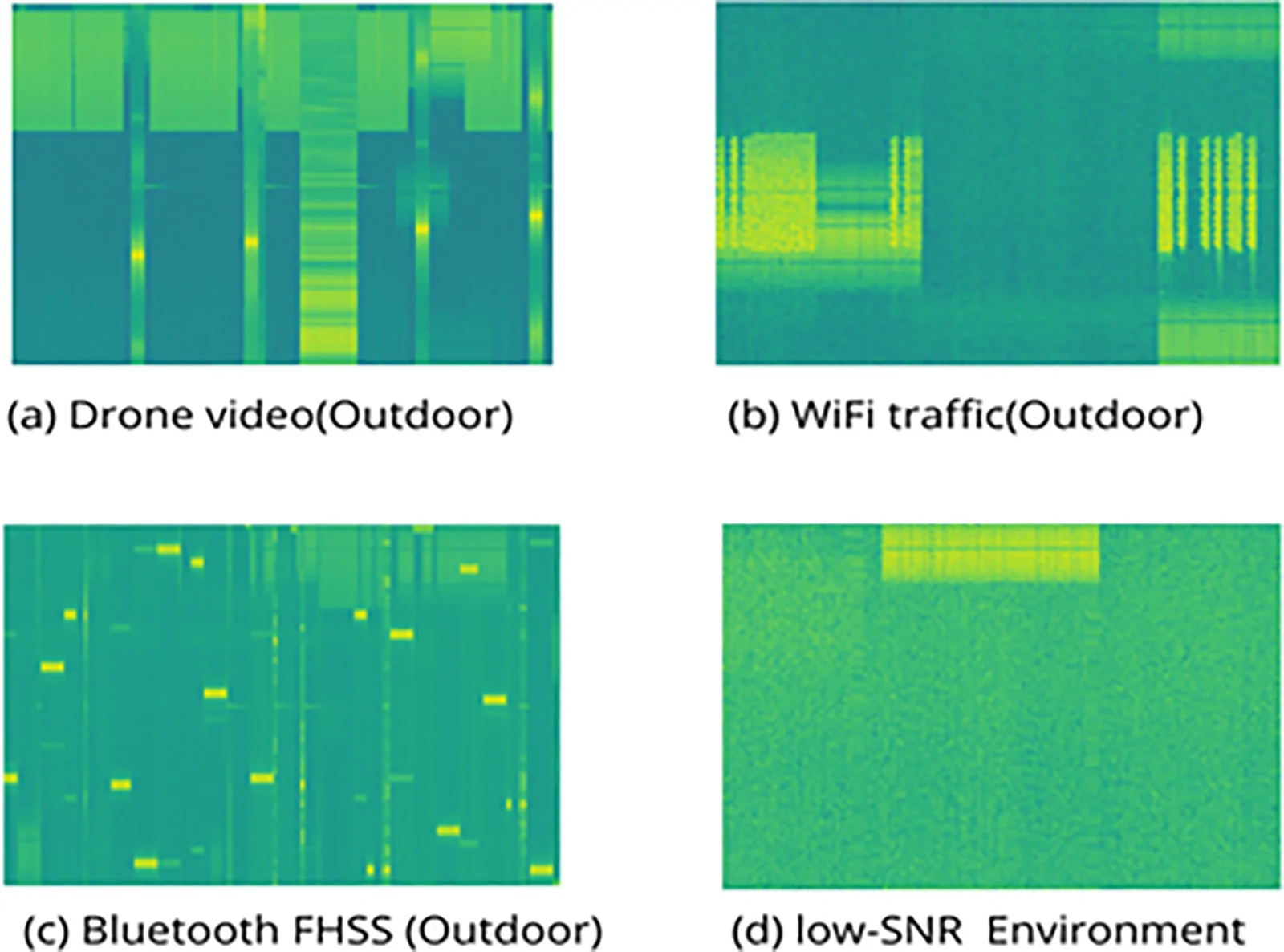
Representative outdoor spectrograms illustrating the impact of uncontrolled environments: (a) Drone video signal showing vertical spectral clutter; (b) WiFi traffic with characteristic wide-band blocks; (c) High-density Bluetooth frequency-hopping bursts; (d) A low-SNR capture where the signal is partially obscured by a raised noise floor.

In contrast, the outdoor spectrograms (illustrated in Fig. 4) present a more complex RF background characterized by the following features:

• Raised Noise Floor and Spectral Clutter: Outdoor acquisitions show a higher level of ambient noise and “clutter” across the 2.4 GHz band. This is a result of cumulative interference from distant residential WiFi access points and multiple uncoordinated consumer electronics.
• Signal Fading and Multipath Effects: Unlike the stable indoor captures, outdoor drone signals may exhibit slight variations in intensity and spectral thickness. This reflects real-world propagation challenges such as multipath fading and signal attenuation over distance.
• Overlapping Interference: In outdoor settings, the density of WiFi and Bluetooth bursts is often higher. This leads to frequent spectral “collisions” where a Bluetooth burst or WiFi packet may temporally overlap with the Drone_Signal, requiring the object detection model to distinguish between overlapping bounding boxes.

By including both clean indoor signatures and cluttered outdoor signatures, the dataset provides a comprehensive benchmark for training deep learning models that are capable of generalizing across different deployment scenarios.

### 4.6 Signal processing and spectrogram generation

The raw complex IQ samples captured by the USRP B210 were transformed into time–frequency representations using the Short-Time Fourier Transform (STFT).

The STFT was computed using the following parameters:

• FFT size (NFFT): 512
• Overlap (noverlap): 256

The time–frequency representation is given by Eq. 1:

(1) $$X\left(t,f\right)=\sum_{n=-\infty }^{+\infty }X\left[n\right]W\left[n-t\right]e^{-j2\pi fn}$$

where x[n] denotes the captured RF signal and w[n] is the analysis window function.

The resulting spectrograms were exported as image files and used as the primary data modality for subsequent annotation and machine learning tasks.

### 4.7 Justification of spectral colormap selection and data augmentation strategy

In the development of RF-based object detection datasets, the choice of time-frequency colormapping is a critical hyperparameter that directly influences the convergence and accuracy of Convolutional Neural Networks (CNNs) [8]. To optimize our dataset for YOLO-based detection, the **Viridis** colormap was selected as the primary visual representation for all raw captures.

**Fig. 5:**
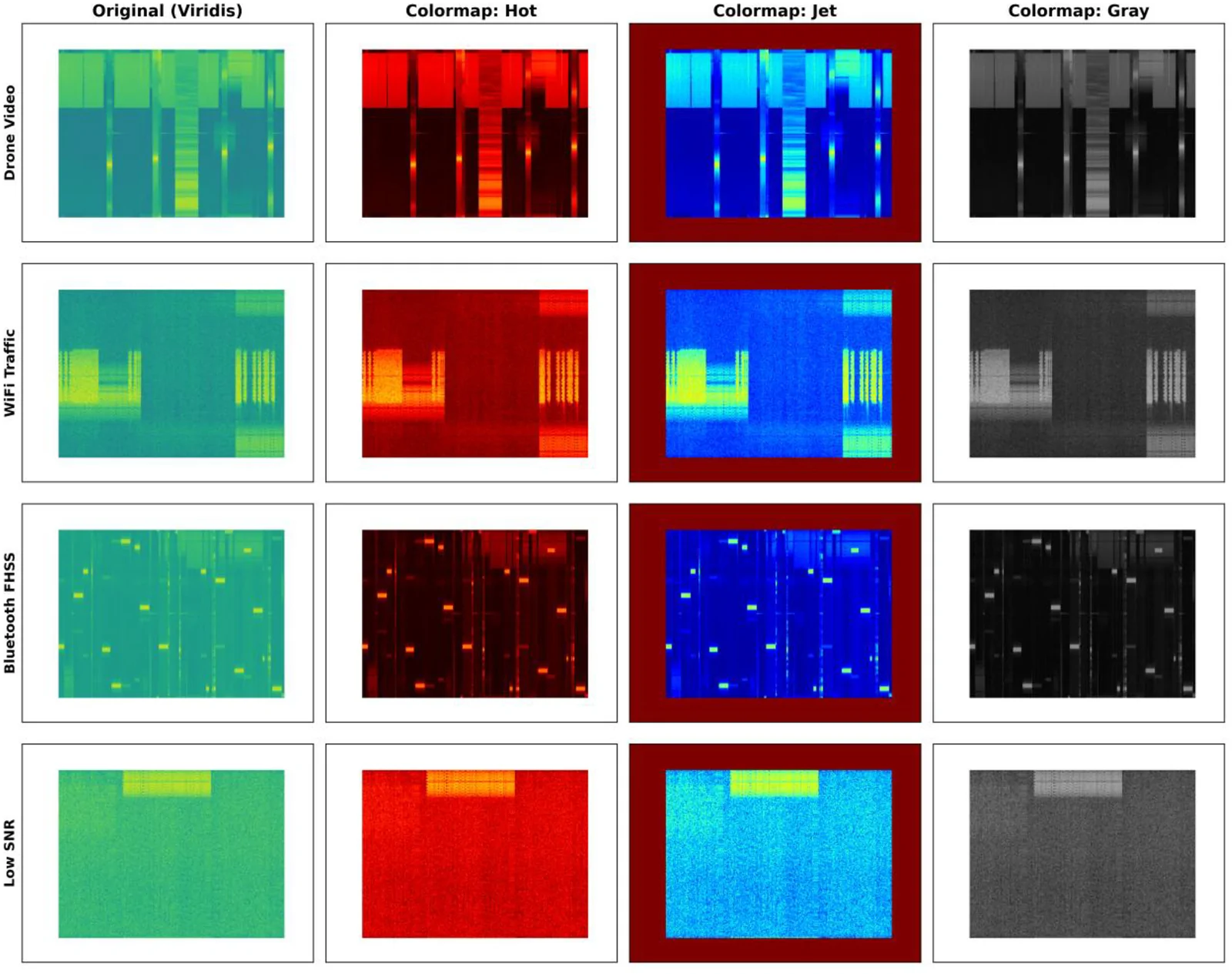
Comparison of the primary Viridis dataset representation against alternative colormaps used for training-time data augmentation. The comparison demonstrates the superior boundary definition of Viridis in uncontrolled outdoor environments.

The necessity of this selection is illustrated in Fig. 5, which provides a comparative analysis of the four target scenarios (Drone, WiFi, Bluetooth, and Low SNR) across various mapping filters. The technical justifications for prioritizing the Viridis mapping are as follows:

• Linear Luminance and Perceptual Uniformity: Unlike the Jet colormap, which exhibits non-monotonic shifts in luminance (creating “false edges” and visual artifacts), Viridis is perceptually uniform. This ensures that the spatial gradients detected by the YOLO architecture correspond directly to actual power density variations in the RF signal, rather than artifacts of the visualization process.
• Dynamic Range in Low-SNR Conditions: As observed in the bottom row of Fig. 5, high-saturation maps like Hot and Jet tend to “clip” or saturate when the noise floor rises in outdoor environments. Viridis preserves a higher dynamic range, allowing the model to distinguish faint drone video signals from background spectral clutter.
• Annotation Precision: The high contrast provided by the Viridis spectrum was essential during the manual labeling phase. It enabled the definition of highly accurate bounding boxes, particularly in complex cases where Bluetooth FHSS bursts temporally overlap with wide-band WiFi or drone signals.

Furthermore, while the base dataset is provided in the optimized Viridis format, our training pipeline incorporates a Spectral Data Augmentation strategy. During the training phase, the original images are dynamically remapped into Hot, Jet, and Grayscale formats. This prevents the model from overfitting to a specific color palette and ensures that the trained weights are robust to variations in hardware visualization or different Software Defined Radio (SDR) software defaults.

### 4.8 Annotation strategy and labeling protocol

The annotation process was designed to generate a high-quality labeled dataset compatible with object detection frameworks while preserving the physical interpretability of the RF signal signatures.

All generated spectrogram images were manually annotated using the LabelImg software [2]. The annotation task was performed at the object level by visually identifying the time–frequency signatures corresponding to each wireless source and assigning the appropriate class label.

Three signal classes were defined for the dataset:

• Drone_Signal: RF emissions associated with the communication link between the DJI Phantom 4 Pro and its remote controller.
• WiFi: wideband wireless local area network activity in the 2.4 GHz ISM band.
• Bluetooth: narrowband frequency-hopping emissions generated by Bluetooth devices.
• The annotation strategy relied on the physical morphology of the spectral signatures observed in the spectrograms.
• Drone_Signal instances were labeled based on their persistent and structured spectral footprint associated with UAV command and telemetry transmissions.
• WiFi signals were identified as relatively wideband and continuous spectral occupancy regions.
• Bluetooth signals were labeled as short-duration and frequency-hopping bursts distributed across the observed bandwidth.

For each detected signal instance, a rectangular bounding box was manually drawn to tightly enclose the visible energy distribution in the time–frequency plane.

Special attention was given to multi-source coexistence scenarios, where multiple signal classes could appear simultaneously within the same spectrogram image. In such cases, each signal source was independently annotated using separate bounding boxes.

The final annotations were exported in YOLO format, where each label file contains:

• class identifier
• normalized center coordinates (x, y)
• normalized width
• normalized height

This labeling strategy ensures direct compatibility with YOLO-based deep learning models and enables supervised training for interference-aware UAV signal detection.

## 5 Data Validation and Robustness Analysis

To rigorously evaluate the proposed YOLOv8-based detection framework [9], validation was conducted across two primary dimensions: quantitative classification accuracy via a normalized confusion matrix, and qualitative model robustness against extreme visual perturbations, specifically varying spectrogram colormaps.

### 5.1 Quantitative validation: confusion matrix analysis

**Fig. 6:**
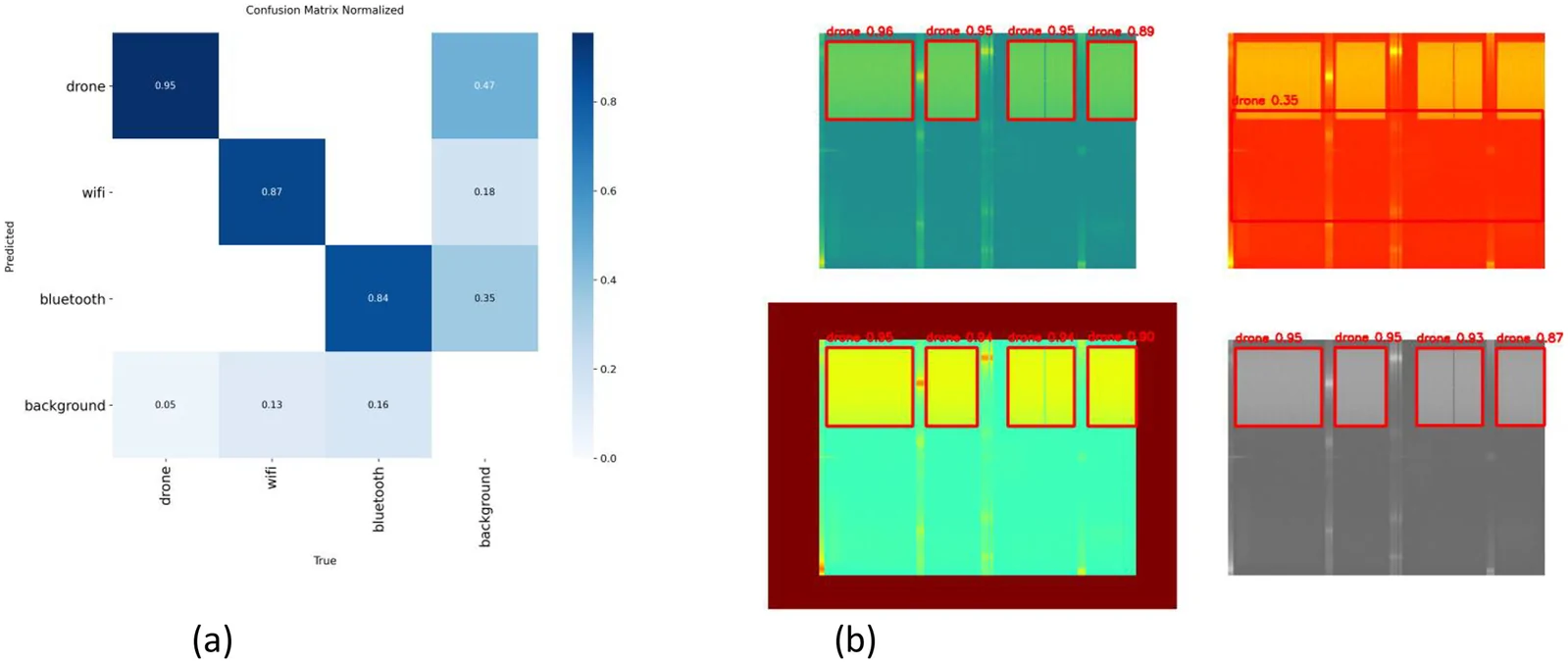
Validation analysis of the proposed RF-UAV detection framework: (a) normalized confusion matrix showing quantitative classification performance; (b) qualitative model robustness against spectral colormap variations.

The overall classification performance of the model is illustrated in the normalized confusion matrix (Fig. 6a). The model demonstrates high discriminatory capability across all active Radio Frequency (RF) classes, achieving exceptional True Positive Rates (TPR).

• UAV Signal Detection: The model achieved a peak accuracy of 95% for drone signal detection. This high accuracy indicates that the multi-task architecture successfully isolates the unique spectral signatures of UAVs from environmental noise. Only 5% of drone signals were misclassified as background (missed detections), highlighting the model's reliability in continuous monitoring scenarios.
• Interference Classification (WiFi & Bluetooth): The model accurately classified transient and wideband interference signals, achieving 87% accuracy for WiFi traffic and 84% for Bluetooth (FHSS) signals. The slightly lower accuracy for Bluetooth compared to the drone class can be attributed to the highly transient, frequency-hopping nature of FHSS bursts, which can occasionally blend into the background noise floor (resulting in a 16% missed detection rate).
• False Positive Distribution: The rightmost column indicates the distribution of background noise incorrectly classified as a signal. When the model triggers a false positive, it predominantly leans toward the “drone” class (0.47) or “Bluetooth” class (0.35). This is an expected artifact in RF spectrogram analysis, as sudden noise spikes closely mimic the morphological features of short telemetry bursts or frequency hops.

### 5.2 Qualitative validation: colormap robustness stress-test

In standard RF analysis, spectrograms are generated using arbitrary colormaps (e.g., mapping amplitude to an RGB gradient). To ensure the model learned the underlying geometric properties of the signals rather than merely overfitting to a specific color palette, a colormap stress-test was performed.

Fig. 6 (Left) illustrates the dataset variability across four distinct spectral mappings: Original (Viridis), Hot, Jet, and Grayscale, applied across different SNR profiles and operational modes (Video, Traffic, FHSS). Fig. 6 (Right) demonstrates the model's inference capabilities on a sample drone spectrogram subjected to these transformations.

• Successful Generalization (Viridis, Jet, Grayscale): The model successfully detected the individual drone signal bursts with high confidence scores (> 0.87) across the Viridis, Jet, and Grayscale mappings. The success on the Grayscale image is particularly notable, proving that the model's feature extraction is heavily reliant on edge detection and luminance gradients rather than isolated chromatic data.
• Colormap Saturation Vulnerability (Hot): The testing revealed a vulnerability when using the “Hot” colormap. Instead of detecting distinct signal pulses, the model predicted a single, generalized bounding box with a severely degraded confidence score of 0.35. This failure mode occurs because the “Hot” colormap heavily saturates high-intensity RF regions into pure red and yellow, effectively washing out the fine boundary edges and gradient details that separate closely spaced bursts.

This validation confirms that while the network exhibits strong generalizability to unseen color mappings through aggressive HSV data augmentation, optimal operational deployment should utilize colormaps that preserve structural gradients (such as Viridis or Grayscale) to prevent the “washing out” of closely clustered RF primitives.

## Limitations

The current dataset is limited to a single UAV platform (DJI Phantom 4 Pro) operating in the 2.4 GHz ISM band. This platform was selected because it represents one of the most widely deployed commercial UAVs and its RF communication characteristics are well-documented, making it an appropriate benchmark for initial dataset development. However, the generalizability to other UAV models, frequency bands (e.g., 5.8 GHz), or communication protocols has not been validated. Different UAV platforms employ varying modulation schemes, transmission power levels, and duty cycles, which may produce distinct spectral signatures. Future work should expand the dataset to include multiple UAV platforms, additional frequency bands, and a broader range of environmental conditions to ensure comprehensive coverage of operational scenarios.

Data acquisition was performed in indoor and semi-controlled outdoor environments, which may not fully represent all real-world deployment scenarios (e.g., dense urban canyons, high-altitude environments). The dataset contains signals from a fixed set of interference sources (one WiFi access point and Bluetooth devices in proximity), which may not capture the full variability of real-world spectral congestion. Additionally, the model’s performance is susceptible to colormap saturation artifacts, as demonstrated by the Hot colormap vulnerability in the robustness stress-test. Future work should expand the dataset to include multiple UAV platforms, additional frequency bands, and a broader range of environmental conditions.

## Declaration

The authors declare that this study did not involve human participants, animal experiments, or the collection of data from social media platforms. Furthermore, all Unmanned Aerial Vehicle (UAV) operations conducted during the data acquisition phase were performed in strict compliance with applicable local aviation regulations, as well as all relevant safety protocols governing commercial drone operations and radio spectrum usage.

## Ethics Statement

The authors declare that this work does not involve human subjects, animal experiments, or the collection of data from social media platforms. Furthermore, all Unmanned Aerial Vehicle (UAV) operations conducted during the data acquisition phase were performed in strict compliance with local aviation regulations and safety protocols regarding commercial drone flight and spectrum usage.

## CRediT Author Statement

**Saber Mgannem:** Conceptualization, Data curation, Writing - original draft. **Radhoine Aloui:** Methodology, Validation, Supervision, Writing - review & editing. **Bilel Hamdi:** Investigation. **Sofien Mhatli:** Software. **Ignacio Llamas-Garro:** Writing - review & editing.

## Data Availability

Original DataThe data are available at: DOI: 10.17632/y4w96kzsb5.1.
